# Air Pollution and Aeroallergens as Possible Triggers in Preterm Birth Delivery

**DOI:** 10.3390/ijerph20021610

**Published:** 2023-01-16

**Authors:** Enrico Cocchi, Valeria Bellisario, Francesco Cresi, Claudio Plazzotta, Claudio Cassardo, Consolata Siniscalco, Licia Peruzzi, Roberto Bono

**Affiliations:** 1 Department of Public Health and Pediatrics, University of Turin, 10126 Turin, Italy; 2 Pediatric Nephrology Unit, Regina Margherita Children’s Hospital, 10126 Turin, Italy; 3 Pediatric Residency School, University of Turin, 10126 Turin, Italy; 4 Biostatistics Residency School, University of Turin, 10126 Turin, Italy; 5 Neonatal Intensive Care Unit, Sant’Anna Obstetric Gynecological Hospital, 10126 Turin, Italy; 6 Physics Department, University of Turin, 10125 Turin, Italy; 7 Department of Life Sciences and Systems Biology, University of Turin, 10123 Turin, Italy

**Keywords:** public health, preterm birth, newborn, acute inflammation, delivery, air pollution

## Abstract

Preterm birth (PTB) identifies infants prematurely born <37 weeks/gestation and is one of the main causes of infant mortality. PTB has been linked to air pollution exposure, but its timing is still unclear and neglects the acute nature of delivery and its association with short-term effects. We analyzed 3 years of birth data (2015–2017) in Turin (Italy) and the relationships with proinflammatory chemicals (PM2.5, O_3_, and NO_2_) and biological (aeroallergens) pollutants on PTB vs. at-term birth, in the narrow window of a week before delivery. A tailored non-stationary Poisson model correcting for seasonality and possible confounding variables was applied. Relative risk associated with each pollutant was assessed at any time lag between 0 and 7 days prior to delivery. PTB risk was significantly associated with increased levels of both chemical (PM2.5, RR = 1.023 (1.003–1.043), O_3_, 1.025 (1.001–1.048)) and biological (aeroallergens, RR ~ 1.01 (1.0002–1.016)) pollutants in the week prior to delivery. None of these, except for NO_2_ (RR = 1.01 (1.002–1.021)), appeared to play any role on at-term delivery. Pollutant-induced acute inflammation eliciting delivery in at-risk pregnancies may represent the pathophysiological link between air pollution and PTB, as testified by the different effects played on PTB revealed. Further studies are needed to better elucidate a possible exposure threshold to prevent PTB.

## 1. Introduction

Preterm birth (PTB) indicates infants born before 37 weeks of gestation, thus, they are not fully developed for extra-uterine life. PTB is a public health concern [1] affecting ~10% of total births worldwide, with higher rates in developing countries and numbers that are constantly increasing [2]. In developed countries, PTB represents the main cause of infant mortality [3] and has been associated with significantly impaired health outcomes [4,5,6] and dramatic community costs [7].

Alongside historical and well known risk factors, namely low socioeconomic status, age, ethnicity, tobacco, substance abuse, poor nutritional status, and the presence of birth defects [8], recent epidemiological studies have linked PTB with chemical air pollution [8,9]. While the effects of air pollution on PTB are now known, the timing with which the effects occur is still controversial [10]. Some studies have claimed that early exposure during pregnancy is responsible [11], while other studies have indicated that late exposure is responsible [12,13]. This confusion is probably due to different pollution-induced reactions causing different detrimental effects on pregnancy depending on the fetal developmental stage in which they occur, their duration, and analyzed outcome. In this regard, studies that have focused on definite narrow temporal windows in order to better elucidate specific pollutants, co-factors, and underlying mechanisms of action are lacking [8]. Moreover, PTB is a complex subject in which the long-term nature of fetal development is associated with an acute medical condition that mandates delivery, with different factors potentially playing very different roles in each scenario. PTB delivery is due to conditions that make it impossible to continue the uterine fetal development. The acute factors either prematurely trigger the delivery itself, or are responsible for such a strong fetal suffering that it overcomes the string mortality and morbidity risks associated with PTB. The role of air pollution in PTB has been historically studied through models inherited from other diseases, such as cardiovascular and neurological diseases, that have a peculiar chronic nature [14]. Similarly, pollution-induced chronic inflammation during pregnancy has been proven to cause developmental impairments that predispose to PTB; however, PTB itself is an acute medical emergency, whose main trigger is an inflammatory distress that induces uterine contractions and subsequent preterm delivery [15]. Interestingly, acute inflammation is the main elucidated mechanism contributing to both PTB and air pollutant-related detrimental effects [16,17]. Short-term exposure to air pollution proved to trigger inflammation, detectable through increased inflammatory markers [18], and mice models have revealed how this induced systemic inflammation could lead to acute diseases [19]. Thus, air pollutant exposure can trigger PTB through an acute inflammatory reaction that elicits uterine contractions and subsequent preterm delivery. In this regard, it is well known how acute inflammation induces uterine contractions and PTB [20], and short-term exposure to air pollutants has been demonstrated to induce an upregulation of delivery-involved inflammatory cytokines, such as interleukins (IL-1 and IL-6), and tumor necrosis factor α (TNF-α), with a strong established role in PTB as well [18,21,22]. Interestingly, this air pollution exposure-induced acute inflammation has been proven to be reversible over days and is pharmacologically addressable [23]. All these lines of evidence advocate for both possible preventive and therapeutic approaches to pollutant exposure and PTB, and reinforce the need to better elucidate underlying mechanisms and possible pharmacological targets through studies focused on specific narrow temporal windows, which are currently lacking.

Thus, the aim of the present study is to assess the specific effects of maternal exposure to chemical and biological air pollutants in the narrow window of 7 days before delivery in both preterm and at-term births. This is in order to better elucidate the role of the air pollutants, other co-factors, and the underlying mechanisms acutely associated with preterm delivery.

## 2. Materials and Methods

### 2.1. Data Collection Area

Turin, the capital of the Piedmont region (North-Western Italy), is one of the most polluted European cities, located 239 m above sea level (a.s.l.); it has 886,837 inhabitants, a population density equal to 6813 per km^2^. Daily data for the period from 1 January 2015 to 31 December 2017 (1096 days) for the city of Turin were collected or derived as described below. The locations of the data sources are shown in Supplementary Materials.

### 2.2. Study Population

Daily data on births were obtained from the birth registry and registration records of the main obstetric hospital in Turin, Italy (Fetal Maternal Department, Sant’Anna Obstetric Gynecological Hospital), recording maternal and fetal data during the 3-year period from 1 January 2015 to 31 December 2017. Recorded data included:

1. Maternal age at delivery;
2. Gestational age (weeks of amenorrhea);
3. Apgar 1/5;
4. Sex;
5. Twins;
6. Mother’s country of origin.

Data were anonymized with respect to the European General Data Protection Regulation (GDPR 101/2018).

### 2.3. Meteorological Data

Meteorological data were obtained from a station placed at 254 a.s.l., on the roof of the Department of Physics of the University of Turin, located at about 2.5 km from the S. Anna Hospital. The station is permanently active and collects weather data in the urban surface layer of the city. Data were collected every 5 s and subsequently averaged every 5 min. Data were aggregated in a daily form for the analysis.

### 2.4. Chemical Air Pollution Data

We extracted concentrations of air pollutants (NO_2_, PM2.5, and O_3_) from data collected at the urban background monitoring station located in Turin, viale Augusto Monti. The data collection and summary was performed by the Local Environmental Protection Agency (ARPA Piemonte), which is coordinated by the regional air pollution service of Piedmont Region, as per the current European legislation (DIR 2008/50/ECX). Data were collected hourly and daily aggregated for the analysis.

### 2.5. Aeroallergen Data

Corylaceae, Cupressaceae, Gramineae, Urticaceae, Ambrosia, and Betula pollens were considered in this study. The measurements of pollen daily data were conducted in a station located ~12 m above the ground without surrounding obstacles, as required in such cases, on the flat roof of a building located 2.6 km from S. Anna Hospital. The sampling station consisted of a HIRST sampler. The HIRST sampler consists of three core parts: a swivel head, a suction pump, and a deposition drum. The latter represents the actual sampling part. This part rotates at 2 mm/h. Collection involved a weekly application of specific adhesive tape on the drum part of the samples. This tape was able to capture aeroallergens, ensuring no loss of rebound or natural detachment. A constant airflow of ~10 L/min was provided by using an air pump, which represented ~14.4 m^3^ daily. Aeroallergen counts were performed by the Department of Life Science and System Biology of the University of Turin, and expressed in our analysis in the form of grains/m^3^.

### 2.6. Statistical Analysis

We summarized quantitative variables as means  ±  SD, medians ± interquartile ranges (IQR), showing minimum and maximum values as well. To allow comparison of variability among different variables, the interquartile ratio (as IQR/median ratio) was also computed. The normality of data was checked through the Kolmogorov–Smirnov test. Quantitative parameters were found to be non-normally distributed, thus, non-parametric tests, such as the Mann–Whitney U-test, served to assess between-group differences for those variables. Group comparisons were computed through chi-square or Fisher’s exact test, as appropriate, based on categorical data under analysis. A two-sided *p*-value < 0.05 was considered to be significant. Pearson’s r coefficient was used to investigate correlations among exposure variables. In order to focus on a narrow temporal window and to correctly assess short-term effects, the associations between PTB (the dependent variable) and concentrations of chemical and biological air pollutants (independent variables) were analyzed using generalized linear models (GLMs) fitting a non-stationary Poisson process [24,25]. We used the following model:

$$f\left(\lambda_{t}+1\right)=\alpha +\overset{k}{\underset{i=1}{\sum }}\beta Xi+NS\left(Z_{t}\right)$$

where

- ƒ: log link function
- *λ_t_*: count of daily PTB at day *t*
- *α*: intercept constant
- *β*: estimated parameters vector
- *X_i_*: matrix of *k* independent variables (exposure and adjustment variables)
- *NS*(*Z_t_*): natural spline smoothing function of calendar day *Z*

As in several literature reports that have focused on the same analysis model, in order to take the medium-/long-term trend that may shape the time data under analysis into account, a natural spline-smoothing function was calculated on 14 degrees of freedom (df) [25]. We restricted possible df to a maximum of 18 (corresponding to a window of ~60-days) in order to avoid overfitting [26,27]. We identified the best df as the value that minimized the absolute values of the residuals partial autocorrelation function (PACF) sum [26]. PACF residuals were corrected for the day of the week, as clarified in the point below, to remove the 7-day positive correlation, when estimating the spline-smoothing function.

We performed further variable adjustments in order to take into consideration characteristics of the location under study that might otherwise bias the model under analysis:

(a) Day of the week (from Monday to Sunday);
(b) Holidays, we considered main holidays in our zone, i.e., Christmas and Easter, ±3 days around them, other holidays, and other days. This resulted in a 4-level variable that was used in the model.
(c) Summer population decrease, in our specific zone, population is known to decrease during summer holidays and this variable was intended to adjust for this effect, resulting in a categorical variable with factors considering such dates, i.e., from Saturday before Mid-August to the next Sunday (for a total amount of 16 days per year), from 16 July to the end of August (removing the aforementioned period), and all other days [28];
(d) Daily average daily temperature (°C);
(e) Daily average humidity (%)—relative;
(f) Daily precipitations (mm)—cumulative.

Once the model was fitted to the actual data, we considered one chemical pollutant among PM2.5, NO_2_, O_3_, or aeroallergens, alongside medium-/long-trend function, non-meteorological variables (day of the week, holidays, and summer population decrease), and meteorological variables (daily temperature, daily relative humidity, and cumulative daily precipitations). Temperature and humidity underwent natural splines with 1 and 2 df transformation, respectively. The df was chosen through PACF criterion as explained above. Daily precipitations were binary coded, i.e., 1 if cumulative precipitation ≥ 1 mm and 0 otherwise.

Exposure variables were included in all the models at different single time lags: starting from the same day of the PTB evaluation (Lag 0) to 7 days before (Lag 7). We identified such a time frame in order to focus the analysis on specific short-term potential effects of air pollutants in eliciting preterm vs. full-term births. To the best of our knowledge, this is the first literature report of such an acute effect-focused analysis. Thus, no previous specific time frame is available. The identification of such a threshold was based on a pathophysiological rationale, as our analysis was based on investigating acute inflammation potential roles in delivery and preterm vs. full-term birth compensation capacities. Thus, as acute inflammation is a process known to exert its effect within a few days from the exposure, we arbitrarily selected this specific 7-day time frame in order to include in the analysis lag all potential acute inflammatory effects of air pollutants, from 24 h to a week from exposure. Associations between exposure variables and preterm or full-term births are reported as relative risk (RR) with respective 95% confidence intervals (CIs). RR values and their CIs are calculated as exponential of GLM resulting coefficients for each specific exposure variable under analysis. Exposure variable association coefficients were calculated based on 10 μg/m^3^ increase in PM2.5, NO_2_, and O_3_ concentrations, and a 10 grains/m^3^ increase in the case of aeroallergens.

## 3. Results

### 3.1. Population Enrolled

In the study period, 21,509 births were observed, of which 3167 PTBs (14.7%). Median gestational age was 39 + 1 for the whole cohort, 39 + 3 for term, and 35 + 1 for PTB. The peculiar differences between preterm and term subpopulations were:

- Higher twins prevalence in PTB (preterm 42.2% vs. term 1.87%, *p* < 0.01);
- Lower Apgar 1/5 scores in PTB (preterm Apgar 1 score 7.6 ± 2.1 vs. term 8.8 ± 0.9, *p* < 0.01; preterm Apgar 5 score 8.2 ± 1.5 vs. term 8.9 ± 0.6, *p* < 0.01);
- Higher maternal age in PTB (preterm 34.1 ± 5.5 vs. term 33.3 ± 5.4, *p* < 0.01).

Cohort characteristics are summarized in Table 1.

### 3.2. Temporal Distribution of Births

**Figure 1:**
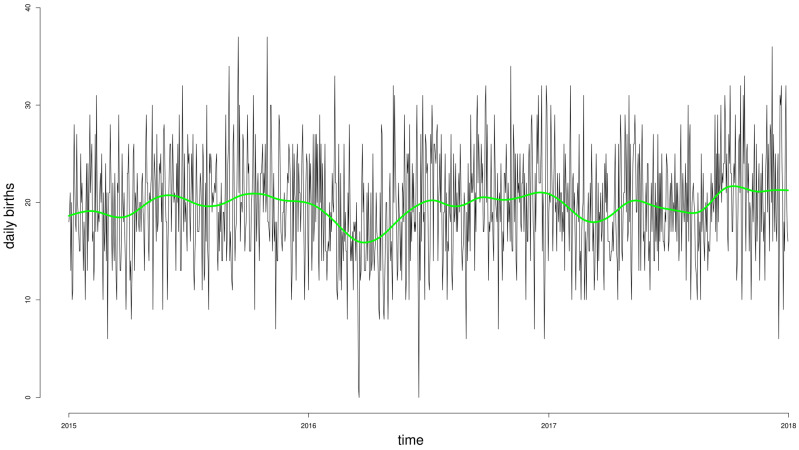
Daily incidence of births during the period under analysis, and the natural spline smoothing function of calendar day *Z* with 14° of freedom utilized in the Poisson non-stationary models (green line).

The temporal distribution of births was analyzed in order to identify the best smoothing strategy possible. Figure 1 graphically reports the daily number of births in the period under study (1 January 2015–31 December 2017) and the natural spline smoothing function of calendar day *Z* with 14° of freedom utilized in the models.

**Figure 2:**
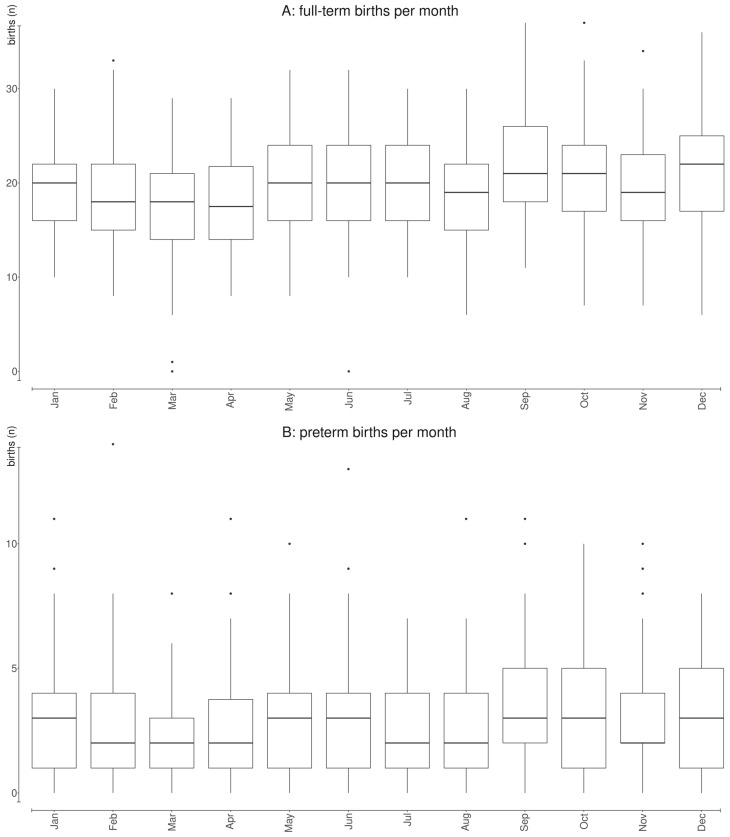
Average number of daily births (**A**) and preterm births (**B**) by month of the year during the 3-year period under analysis (1 January 2015–31 December 2017).

Figure 2 summarizes the average number of daily births (Figure 2A) and PTBs (Figure 2B) by month of the year, revealing the same unclear pattern of seasonality detectable in daily data in Figure 1 and addressed through the spline smoothing function. 

### 3.3. Environmental Pollutants and Allergens

Table 2 shows a general description of the daily concentrations of chemical and biological air pollutants in the area under study during the 3 years examined.

**Figure 3:**
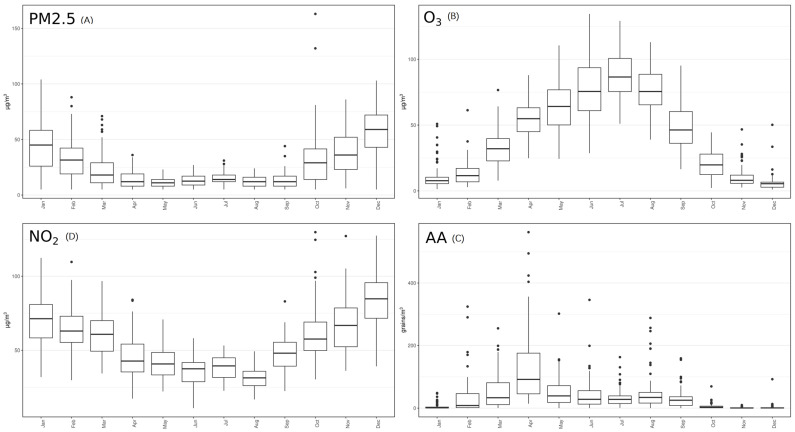
Mean concentration of each pollutant analyzed by month during the 3-years period under analysis. AA: aeroallergens.

Chemical pollutants show rather high concentration levels, in accordance with the situation already studied in this area [24,28]. Due to their natural seasonality, aeroallergen concentrations showed larger variability as compared with chemical air pollutants (even if chemicals also have a seasonality dependent on their primary and/or secondary origin). According to the origin, pollutants are categorized as primary and secondary. Primary pollutants are directly emitted from their sources, while secondary pollutants are either formed by the atmospheric transformation of primary pollutants or other chemical compounds. PM2.5, and NO_2_ (Figure 3A,D) showed a prevailing maximum level during the coldest months, as expected due to its primary origin. Contrarily, O_3_ showed a behavior typical of a pollutant of secondary origin (Figure 3B). Aeroallergens showed high concentrations during warm season, and were virtually absent in winter (Figure 3C), as expected.

**Figure 4:**
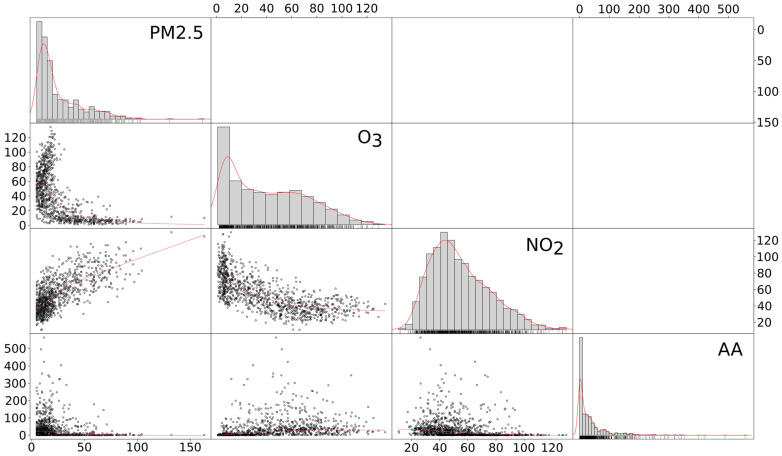
Matrix correlation plot among pollutants under analysis. The red lines over histograms represent the density function of that variable distribution. The red straight lines in scatter plots represents the correlation r 2 splope between the pollutants plotted in each scatter plot. AA: aeroallergens.

A positive linear association between PM2.5 and NO_2_ was detected (RR = 0.51, *p* < 0.01) and negative associations of O_3_ with PM2.5 (RR = −0.34, *p* < 0.01) and NO_2_ (RR = −0.46, *p* < 0.01). The associations between chemical pollutants and aeroallergens were weaker but still significant (PM2.5, RR = 0.06, *p* < 0.01; NO_2_, RR = 0.04, *p* < 0.01; O_3_ RR = 0.08, *p* < 0.01). All correlation plots are reported in Figure 4.

### 3.4. Correlations between Birth and Environmental Data

Potential confounders and their relationships were considered before the analysis. Table 1 and Table 2 summarize the mean number of daily births, air pollution concentrations, and aeroallergen concentration according to the considered potential confounders. PTBs were less frequent during weekends and holidays than during the other days. PM2.5 and NO_2_ were inversely associated with temperature, due to their secondary component nature, whose synthesis is favored by the sun. O_3_ and aeroallergens were positively associated with temperature, as expected. Airborne pollution was lower during rainy days, as expected. 

#### 3.4.1. Preterm

**Figure 5:**
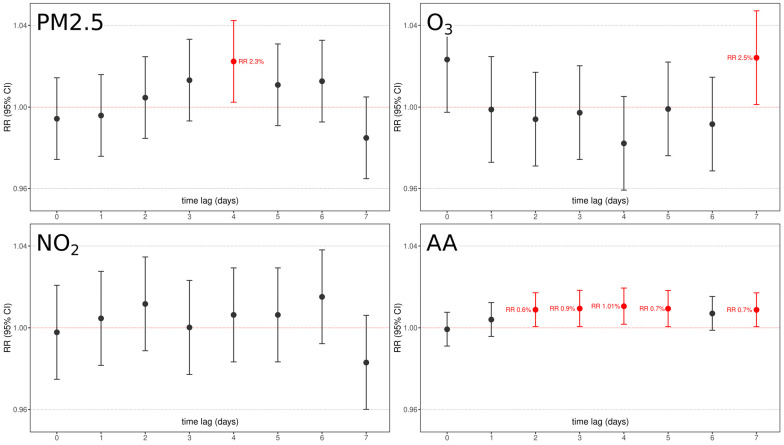
Graphical representation of chemical pollutants under analysis (PM2.5, NO_2_, O_3_) and aeroallergen (AA) concentrations relative risk and confidence interval association with PTB as per 7-days time lags. Red boxes underline significant factors with their relative lag RR. All models were adjusted for day of the week, holidays, summer population decrease, and yearly medium/long-term trend function.

The associations between exposure variables and PTB through the Poisson model adjusted for all potential confounders (day of the week, holidays, summer population decrease, daily temperature, daily relative humidity, and cumulative daily precipitations), are summarized in Figure 5. 

With the exception of NO_2_, all pollutants were significantly associated with PTB at some time lag:

(1) An increase of 10 μg/m^3^ of PM2.5 is associated with a significant 1.023 (RR 95% C.I. 1.003–1.043, *p* < 0.05) increased risk of PTB after 4 days.
(2) An increase of 10 μg/m^3^ of O_3_ is associated with a significant 1.025 (RR 95% C.I. 1.001–1.048, *p* < 0.05) increased risk of PTB after 7 days.
(3) An increase of 10 grains/m^3^ of aeroallergens is associated with an increased risk of PTB from 2 to 7 days after exposure:
  - 1.006 (RR 95% C.I. 1.002–1.012, *p* < 0.05) after 2 days;
  - 1.009 (RR 95% C.I. 1.002–1.014, *p* < 0.01) after 3 days;
  - 1.01 (RR 95% C.I. 1.004–1.016, *p* < 0.01) after 4 days;
  - 1.007 (RR 95% C.I. 1.001–1.013, *p* < 0.04) after 5 days;
  - 1.007 (RR 95% C.I. 1.0008–1.03, *p* < 0.05) after 7 days.

The characteristic lag distribution effects that are detectable for aeroallergens, i.e., either the significant effect detectable at lag7 or the not significant detectable at lag6, may be related to the specific cohort under analysis. The results obtained suggest that aeroallergens are likely to play a role in eliciting PTB, and this role is probably played over different days, probably underlying a stronger proinflammatory effect on some specific target, according to their characteristics. Further analysis with larger, more different cohorts, and more years of data collection are needed in order to better elucidate the real significant nature and lags of aeroallergens on PTB.

#### 3.4.2. Term

**Figure 6:**
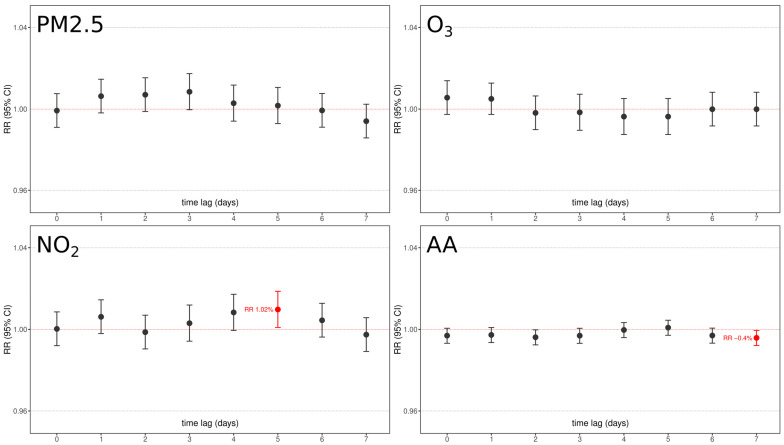
Graphical representation of chemical pollutants under analysis (PM2.5, NO_2_, O_3_) and aeroallergen (AA) concentrations relative risk and confidence interval association with full-term births as per 7-days time lags. Red boxes underline significant factors with their relative lag RR. All models were adjusted for day of the week, holidays, summer population decrease, and yearly medium/long-term trend function.

The correlation analysis between at-term delivery and chemical pollutants and aeroallergens was performed in the exact same way as for PTB and the results are summarized in Figure 6. 

Essentially, the results showed that no pollutant was significantly associated with an increased risk of term delivery, with only two barely significant associations: an increase of 10 μg/m^3^ of NO_2_ resulted in an increased risk of 1.01 (RR 95% C.I. 1.002–1.021, *p* < 0.05) after 5 days from exposure, and an increase of 10 grains/m^3^ of aeroallergens resulted in a very fleble protective effect after 7 days from exposure (RR 0.996, RR 95% C.I. 0.999–0.993, *p* < 0.05). No other air pollutants were associated at any time lag.

## 4. Discussion

Air pollution impact on PTB is well known and has been variously assessed, but specific time-frame evaluation is still lacking. The peculiar and complex nature of PTB, where a long-term process (fetal development) is paired with an acute one (preterm delivery) needs tailored models that are focused on specific outcomes and confounders based on the period considered. While PTB has normally been assessed with models historically developed for chronic diseases, no comprehensive model has been applied for correctly assessing the short-term effects of air pollutants in a narrow temporal window before delivery. The complex nature of PTB may lead to confusion in the interpretations of the different risk effects identified through epidemiological studies, as reported. In fact, while the assessment of long-term effects is of certain importance in order to elucidate the influence of prolonged exposures in fetal development and subsequent predisposition to PTB, the assessment of short-term effects associated with actual delivery may pave the way to easier and more effective interventions immediately applicable to prevent PTB. Long-term changes would require radical and prolonged lifestyle changes, while short-term exposure preventive measures could be performed more easily and immediately. Moreover, air pollution levels are monitored widely and continuously in developed countries and the data are easily retrievable, providing important information that may help in identifying acute exposure thresholds [29,30,31], especially for at-risk pregnancies.

Regarding the regression model selection, as presented above, our analysis was specifically aimed at investigating the precise acute effects of air pollution on potentially eliciting preterm vs. full-term birth. This acute focus is the core behind model selection. The graphical analysis of our data and the time distribution of daily births showed that the variable is best approximated by Poisson distribution, with a higher λ in the case of at-term births. Thus, a generalized linear Poisson model was considered to be the best model for the analysis, according to previous literature reports that had focused on the same acute effects under investigation [24,25,26]. On the one hand, the results of this specific analysis approach show that a specific role of some specific proinflammatory air pollutants in eliciting preterm births is detectable. On the other hand, this acute inductive effect of air pollution is undetectable in the case of full-term births, in which we identify different effects of the various pollutants under analysis. According to the aim of our analysis, this difference may reflect some addictive effects of pregnancy-related pathological conditions that are brought to delivery by air pollution-related stress in the case of preterm births, while the same effects are likely compensated for in physiological full-term births.

Our results show that air pollutants could play significant roles on PTB in the days immediately preceding delivery. In the 7 days preceding PTB delivery, an increased exposure to airborne chemicals and aeroallergens is detectable. An exposure to PM2.5, 5 days preceding PTB, increases its risk by 2.3% for every 10 μg/m^3^ PM2.5. In a similar manner, 7 days before PTB, an exposure to O_3_ increases its risk by 2.5% every 10 μg/m^3^. Fascinatingly, an increased exposure of 10 grains/m^3^ of aeroallergens showed an almost continuous (from day 2 to day 7) increase of risk of ~1% on PTB, further testifying their strong inflammatory nature that has been revealed in asthma and other diseases. Interestingly, none of these effects were detectable on physiological term delivery. Contrarily, aeroallergens resulted in a weak but protective effect, and NO_2_ that showed no effect on PTB resulted in an increased ~1% risk of delivery every 10 μg/m^3^ at 5 days time lag. This fascinating difference between PTB and term births may testify the different trigger effects that these pollutants play in at-risk vs. physiological pregnancies, where the latter might have enough resources to cope with this proinflammatory noxa. This difference may be related to molecular differences in inflammatory pathways elicited by these pollutants on different physical states, whose deeper exploration is mandatory to better clarify PTB pathophysiology. Our analysis is corrected for all possible environmental, seasonal, and social confounding factors that frequently bias these analyses, as testified by the significant differences in PTB vs. term incidence once grouped by day of the week, holidays, population decrease, and temperature. 

Due to the huge number of individuals exposed, air pollution may explain an important portion of cases, and allow preventive strategies. For example, consider an annual mean concentration of PM2.5 of ~25 μg/m^3^ in the Turin area [32] versus 10 μg/m^3^ in nearby Switzerland, this single difference can lead to an increased ~5% risk of PTB only attributable to PM2.5 levels. Considering ~1050 PTB cases per year, this represents ~50 PTB cases only attributable to PM2.5 exposures during days immediately preceding delivery.

The focus on a narrow time frame before delivery is of core importance for both the abrupt nature of preterm delivery and applicable potential preventive measures. For example, a strong recommendation for at-risk women to avoid highly polluted areas in the last trimester of gestation may easily save several newborn lives and may avoid PTB-related complications. Moreover, as previously explained, Il-1, IL-6, and TNF-a, among other inflammatory cytokines, exert a central role in PTB and are increasingly produced as a consequence of air pollution exposure. In this regard, pollutant-induced acute inflammation may offer the molecular explanation for this relationship, and inflammation can also be effectively and safely monitored through these biomarkers.

Study limitations: The limitations of the current studies are mainly related to the single-center nature of the study and the highly polluted area considered. In fact, the area under analysis is a particularly polluted area, and thus, a good candidate in order to elucidate the possible role of air pollution exposure on PTB, but RR and exposure effects may vary in other areas. The single-center nature of the study is also associated with the absence of spatial resolution, as only one monitor served for all patients under analysis. Moreover, air pollutants have been recorded in a single site relatively close to the hospital, which is the only one available with such precise measurement. We considered such measurements as indicators for the overall area under analysis, even if a measurement of each woman’s living place would lead to a much more accurate analysis. Another main limitation of the current study is the absence of laboratory testing for inflammation markers in the cohort under analysis, as this was intended as an exploratory analysis in order to confirm/exclude possible roles of such molecules on PTB. Inflammation marker testing is currently being performed on a subcohort of the presented one, as a continuation of the current work. The multiple comparison of lag times could result in a type I error; to be consistent with the same model presented in our previous work [24] we did not correct for Bonferroni, in order to avoid exclusion of interesting trends. Nonetheless, the Bonferroni threshold over the 7-day lag comparison would set a 0.007 *p*-value threshold.

## 5. Conclusions

Our findings suggest that a specific role of some specific proinflammatory air pollutants on delivery induction is detectable in PTB, while lower and different direction effects are detectable in full-term births. This fascinating difference advocates for extensive studies on air pollutant proinflammatory pathophysiology mechanisms and their specific relationships with PTB. In this view, further identification of physiological differences underlying such relationships may help to identify potential pharmacological targets that would further help to prevent PTB. Moreover, to the best of our knowledge, our analysis is the first analysis that has specifically focused on acute eliciting effects of air pollutants on PTB vs. full-term births. We were able to detect pollutant effects that have not been previously reported, probably due to their specific proinflammatory short-term effects, which were undetectable in the case of long-term studies that have represented the main approach to date. Thus, in particular, it is important to broaden our time-frame reference when investigating such complex matters, in which an overlap of long- and short-term effects plays a role on the same outcome, elaborating specific models that are able to consider precise time frames to differently investigate chronic vs. acute effects. Finally, the detection of precise short-term acute effects of air pollutants on PTB advocates for the implementation of preventive regulations regarding air quality, especially in urban centers and for at-risk pregnancies. Considering the huge number of pregnant women exposed, it is of central importance to further investigate this relationship and possible preventive thresholds, in order to rapidly and easily apply air pollution-related recommendations for the last months of pregnancies and to further reinforce the beneficial effects of policies aimed at reducing air pollution. These anti-pollution policies may prevent a huge number of PTBs, alongside other countless beneficial effects on other well known and reported aspects of human health. 

## Figures and Tables

**Table 1:** Characteristics of the cohort under analysis.

	All Cohort (*n* = 21,509)				Term (*n* = 18,345)				Preterm (*n* = 3164)			
	Median (IQR)	IQR/Median	Min–Max	Mean ± SD	Median (IQR)	IQR/Median	Min–Max	Mean ± SD	Median (IQR)	IQR/Median	Min–Max	Mean ± SD
Maternal age at delivery (weeks)	33.7 (7.4)	0.2	14.8–48.6	33.4 ± 5.4	33.6 (7.4)	0.2	14.8–48.6	33.3 ± 5.4	34.3 (7.7)	0.2	15.6–46.9	34.1 ± 5.5
Gestational age (weeks + days)	39 + 1 (2 + 1)	0.05	21 + 5–49 + 0	38.7 ± 2.3	39 + 3 (1.7)	0.04	37 + 0–49 + 0	39.4 ± 1.5	35 + 1 (3 + 0)	0.09	21 + 5–36 + 6	34.3 ± 2.7
Apgar 1	9 (0)	0	0–10	8.6 ± 1.2	9 (0)	0	0–10	8.8 ± 0.9	8 (2)	0.2	0–10	7.6 ± 2.1
Apgar 5	9 (0)	0	0–10	8.9 ± 0.8	9 (0)	0	0–10	8.9 ± 0.6	9 (1)	0.11	0–10	8.2 ± 1.5
Sex prevalence (%)	Male 11,066 (51.4%)				Male 9423 (51.4%)				Male 1640 (51.8%)			
	Female 10,443 (48.6%)				Female 8919 (48.6%)				Female 1524 (48.2%)			
Twins prevalence (%)	Single 20,005 (93.1%)				Single 17,999 (98.3%)				Single 1977 (62.5%)			
	Double 1438 (6.6%)				Double 317 (1.7%)				Double 1121 (35.4%)			
	>2: 66 (0.3%)								>2: 66 (2.1%)			
Maternal country of origin prevalence (%)	Italy 16,032 (74.5%)				Italy 13,649 (74.4%)				Italy 2381 (75.2%)			
	Romania 1769 (8.2%)				Romania 1512 (8.2%)				Romania 257 (8.1%)			
	Morocco 768 (3.6%)				Morocco 651 (3.6%)				Morocco 116 (3.7%)			
	Nigeria 440 (2%)				Nigeria 368 (2%)				Nigeria 72 (2.3%)			
	Albany 292 (1.4%)				Albany 252 (1.4%)				Albany 40 (1.3%)			
	Peru 251 (1.2%)				Peru 214 (1.2%)				Peru 37 (1.2%)			
	Egypt 244 (1.1%)				Egypt 218 (1.2%)				Egypt 26 (0.8%)			
	China 152 (0.7%)				China 136 (0.7%)				China 17 (0.5%)			
	Other 1561 (7.3%)				Other 1342 (7.3%)				Other 221 (6.9%)			

**Table 2:** Distribution of daily concentrations of air pollution and aeroallergens during the three years examined (1 January 2015–31 December 2017).

	Available Data (Days)	Median (IQR)	Interquartile Ratio	Min–Max	Mean ± SD
PM2.5 (μg/m^3^)	996	17.0 (24.0)	1.4	5.0–163.0	25.6 ± 21.4
NO_2_ (μg/m^3^)	1081	49.9 (29.6)	0.6	10.9–129.8	54.1 ± 21.0
O_3_ (μg/m^3^)	1083	19.9 (51.1)	2.6	1.7–262.3	41.1 ± 46.6
Aeroallergens (grains/m^3^)	888	11.8 (42.1)	28.1	0.0–563.0	36.0 ± 61.4

## Data Availability

All the raw data collected are available, following a justified request to the corresponding author.

## Supplementary Materials

The following supporting information can be downloaded at: https://www.mdpi.com/article/10.3390/ijerph20021610/s1, Figure S1: The figure geographically illustrates Turin position inside Piedmont region, and Piedmont position in Italy.
