# Supplementary figures and images for: Air Pollution and Aeroallergens as Possible Triggers in Preterm Birth Delivery
Source: Int J Environ Res Public Health. 2023 Jan 16;20(2):1610. doi: 10.3390/ijerph20021610 (PMC9860587; doi:10.3390/ijerph20021610)

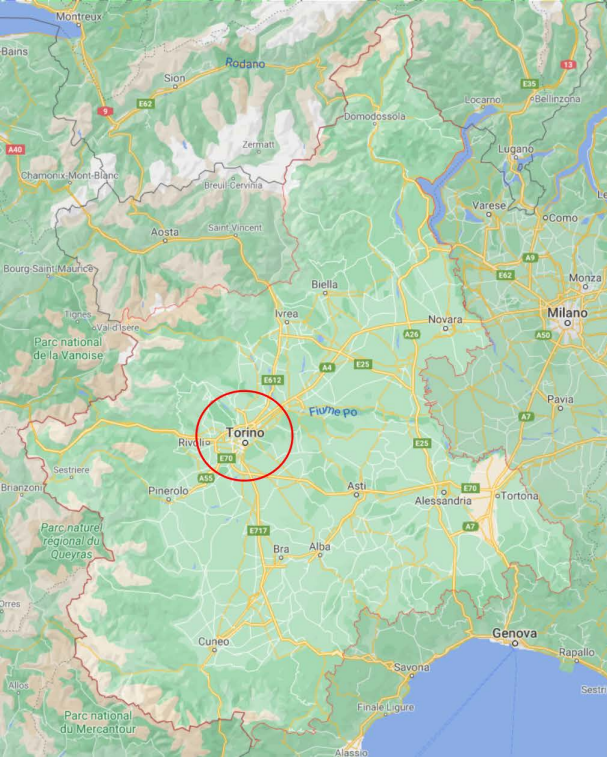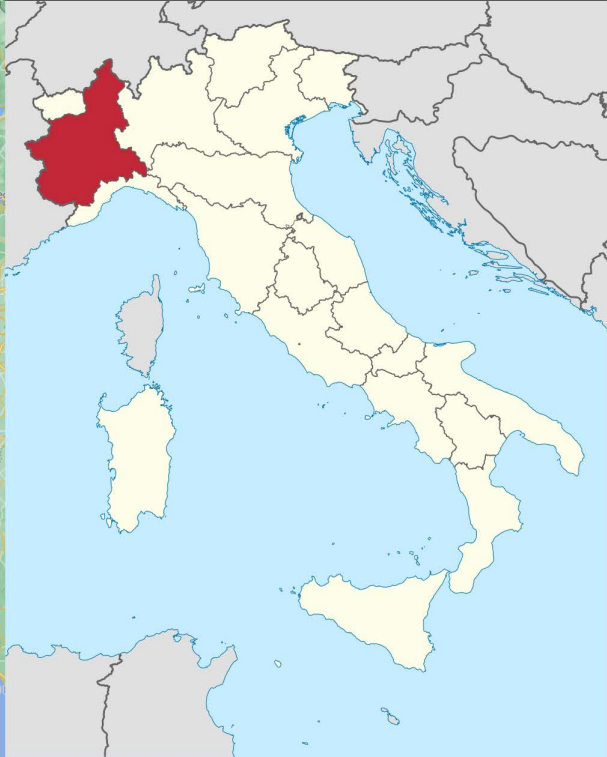

Supplement: Supplementary file 1 [file ijerph-20-01610-s001.zip › ijerph-2067919-supplementary.pdf]
